# Managing End-Of-Life Decision Making in Intensive Care Medicine – A Perspective from Charité Hospital, Germany

**DOI:** 10.1371/journal.pone.0046446

**Published:** 2012-10-01

**Authors:** Jan A. Graw, Claudia D. Spies, Klaus-D. Wernecke, Jan-Peter Braun

**Affiliations:** 1 Department of Anesthesiology and Intensive Care Medicine, Campus Charité Mitte and Campus Virchow Klinikum, Charité – Universitätsmedizin Berlin, Berlin, Germany; 2 Institute of Medical Biometry, Campus Charité Mitte, Charité - Universitätsmedizin Berlin and SOSTANA GmbH, Berlin, Germany; University of Nebraska Medical Center, United States of America

## Abstract

**Introduction:**

End-of-life-decisions (EOLD) have become an important part of modern intensive care medicine. With increasing therapeutic possibilities on the one hand and many ICU-patients lacking decision making capacity or an advance directive on the other the decision making process is a major challenge on the intensive care unit (ICU). Currently, data are poor on factors associated with EOLD in Germany. In 2009, a new law on advance directives binding physicians and the patient´s surrogate decision makers was enacted in Germany. So far it is unknown if this law influenced proceedings of EOLD making on the ICU.

**Methods:**

A retrospective analysis was conducted on all deceased patients (n = 224) in a 22-bed surgical ICU of a German university medical center from 08/2008 to 09/2010. Patient characteristics were compared between patients with an EOLD and those without an EOLD. Patients with an EOLD admitted before and after change of legislation were compared with respect to frequencies of EOLD performance as well as advance directive rates.

**Results:**

In total, 166 (74.1%) of deaths occurred after an EOLD. Compared to patients without an EOLD, comorbidities, ICU severity scores, and organ replacement technology did not differ significantly. EOLDs were shared within the caregiverteam and with the patient´s surrogate decision makers. After law enacting, no differences in EOLD performance or frequency of advance directives (8.9% vs. 9.9%; p = 0.807) were observed except an increase of documentation efforts associated with EOLDs (18.7% vs. 43.6%; p<0.001).

**Conclusions:**

In our ICU EOLD proceedings were performed patient-individually. But EOLDs follow a standard of shared decision making within the caregiverteam and the patient´s surrogate decision makers. Enacting a law on advance directives has not affected the decision making-process in EOLDs nor has it affected population´s advance care planning habits. However, it has led to increased EOLD-associated documentation on the ICU.

**Trail Registration:**

ClinicalTrials.gov NCT01294189.

## Introduction

Mortality on intensive care units ranges from 6–18% in Europe [1]–[5]. The subsequent introduction of multiple artificial organ support and replacement technology has caused a redefinition of death – shifting it from a sudden and unexpected event to a process [6]. The boundaries between medical therapy prolonging life to this therapy prolonging dying became fluid. Consequently most patients in the intensive care unit (ICU) (60%–80%) die after an end-of-life-decision (EOLD) has been made, a decision to limit full life support [1]–[4], [7], [8].

During the last century medical decision making in Europe has shifted from a paternalistic, physician centered approach towards a more patient centered model of shared decision making [9], [10]. In Europe and the US the majority of people favours this concept [9]–[13]. However, this model becomes complicated when a patient loses decision making capacity. Rates of advance directives are generally low in Germany as well as in other countries [1], [4], [5], [14]–[16]. In September 2009 the “advance-directives-law” was enacted in Germany. For the first time a statutory law regulates advance directives. Physicians have to respect a written advance directive of an adult patient in any decision concerning medical treatment. A patients´s attorney or surrogate decision maker is not allowed to overrule a patient´s advance directive as it was possible priorly [17].

However, there is a lack of data describing the daily proceedings and the factors associated with EOLDs in German ICUs [4], [18], [19]. Therefore at first we compared characteristics of patients who received an EOLD with those who received no EOLD. Secondly we studied the incidences of different intensive care therapeutic strategies that were limited.

Finally, as the law for advance directives was established during our observation period, we were able to analyze how those changes of legislation influenced EOLDs on our ICU. We studied this with a special regard to the recommendations for the participation of different decision-makers according to the 5^th^ International Consensus Conference in Critical Care [9].

## Methods

The Medical Ethics Committee of Charité University Hospital approved this study (number of ethical approval EA1/292/10). The study was registered as a clinical trial (ClinicalTrials.gov Identifier: NCT01294189). Informed consent was waived due to the retrospective and observational nature of the study.

### Setting

This retrospective study was performed in a 22-bed surgical Intensive Care Unit led by the Department of Anesthesiology and Intensive Care Medicine at Charité University Medicine. The ICU is covered by in-house consultants with an ICU board certification 24 hours per day, seven days a week. Furthermore, Fellows board certified in anesthesiology and intensive care medicine are available 24 hours a day, seven days a week on the ICU. Additionally, two residents are present on the ICU continuously. Daily rounds involve at least one consultant with board certification in intensive care medicine.

### Patients

**Figure 1:**
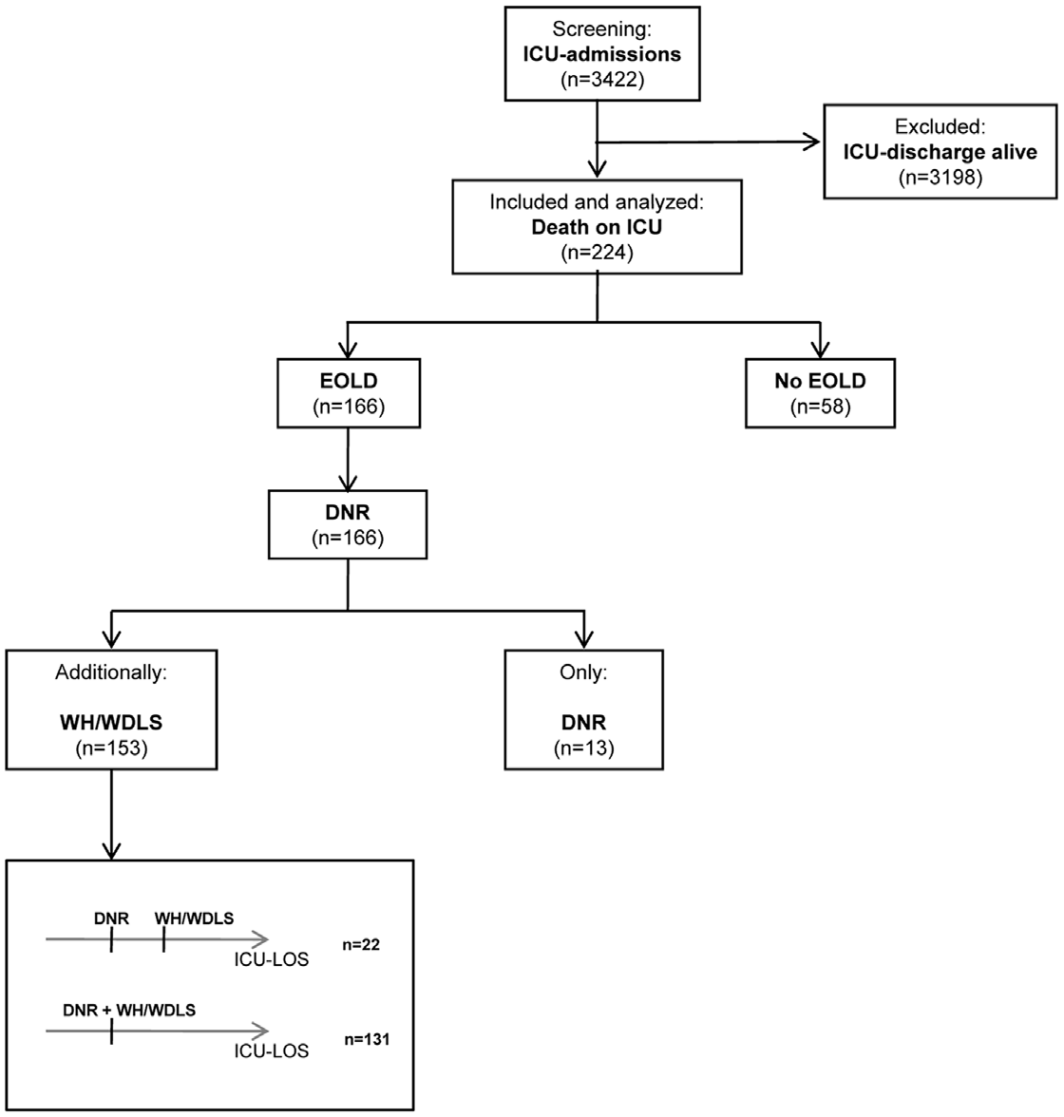
Consort diagram.

The study includes all consecutively admitted ICU patients who died between August 1^st^ 2008 and September 31^st^ 2010. Precisely in the median period of this study the “advance-directives-law” was enacted on September 1^st^ 2009. During the observation period 3422 patients were admitted to the ICU of whom 224 died (6.5%) before of discharge. One hundred sixty-six those patients (74.1%) had an EOLD (Fig. 1).

### Groups

An EOLD was defined as a Do-not-resuscitate (DNR) order, meaning not to initiate cardiopulmonary resuscitation (CPR) in patients who had a cardiac or respiratory arrest. Also any order to withhold and/or withdraw life support (WH/WDLS), meaning intensive care therapeutic approaches that were otherwise warranted, was considered an EOLD. Limitations differentially included withholding or withdrawing therapies like endotracheal intubation, mechanical ventilation, renal replacement therapy, catecholamine infusions, surgery, antimicrobial therapy, blood product transfusions, nutrition and hydration.

We compared all patients with a DNR order to patients without an EOLD with respect to comorbidities, ICU severity scores, organ replacement technology, advance directive rates, and timing of EOLDs or death. We also compared all patients with a WH/WDLS order to patients without an EOLD, respectively.

The day of the EOLD was defined as the calendar day in the ICU on which the decision was made. Comparing time dependent variables between patients who received an EOLD and those who did not receive an EOLD, we set the day of no EOLD as the calendar day in the ICU on which the patient died.

For patients with a WH/WDLS order we analyzed how the differential limitation of therapeutic approaches was performed in detail. Data collected for WH/WDLS orders refers to variables for a patient´s first WH/WDLS order.

Furthermore, we compared patients with an EOLD who were admitted before September 1^st^ 2009 to patients who were admitted thereafter with regard to participation frequencies of members of the caregiver team and the patient´s family in EOLDs, patient and family information and involvement rates, advance directive rates, and frequencies of documentation in an additional, specifically reserved “EOLD-section” of the patients´ records.

As a law needs a time period to be implemented in a second analysis we allowed for a so called “wash-out” period of six months after September 1^st^ 2009.

### Data Collection

Since 2004 the patient records on our ICUs are kept electronically with a Patient data management system (PDMS) (Copra System, Sabachswalden, Germany). The PDMS automatically records data from vital signs monitors, ventilators, organ replacement systems, medication, daily ICU scores like the simplified acute physiology score II (SAPS II) and the sequential organ failure assessment (SOFA), and all medical or nursing events to the patient. Documentation of medical staff, progress notes and orders as well as laboratory results are done complete electronically. The presented data were obtained retrospectively from the daily progress notes, daily vitals signs and medication charts as well as the daily organ replacement and ventilation charts of the PDMS.

Limitations of therapy were documented in the daily progress notes with time and participants of EOLD-conferences by the physician in charge of the patient. Besides the daily progress notes there is a section in the patient records that is specifically reserved for all EOLD-associated documentation. Patients received an EOLD only when every participant of the EOLD-conference consented to the decision and its several regulations.

### Statistical Analysis

Results are expressed as arithmetic mean ± standard deviation (SD) or median with 25%–75% quartiles for continuous variables, and frequencies (%) with 95% confidence intervals (CI) for categorical variables, respectively. Due to the different sample sizes and the skewness of distributions only nonparametric exact tests were applied.

Differences between the regarded groups were tested by the non-parametric (exact) Wilcoxon-Mann-Whitney test for independent groups. Frequencies were tested by the (exact) Chi-square-test in contingency tables. A two-tailed p-value <0.05 was considered statistically significant. All tests were conducted in the area of exploratory data analysis. Therefore, no adjustments for multiple testing have been made. All numerical calculations were performed with *Predictive Analytics SoftWare* (PASW), Version 18.

## Results

EOLDs were taken within a median ICU length of stay (LOS) of five days for DNRs (interquartile range (IQR): 2–15) as well as for WH/WDLS decisions (IQR: 2–19). After any EOLD patients died within a median of one day (IQR: 0–3). The characteristics of the decedents are presented in Table 1. Furthermore differences in baseline comorbidities, ICU severity scores, ICU-LOS, organ replacement technology and advanced care planning for the different groups are shown in Table 1.

**Table 1:** Characteristics of Patients that died on the intensive care unit between August 2008 and September 2010.

	All		No EOLD		DNR		p* ^1^	WH/WDLS		p* ^2^
	n = 224		n = 58		n = 166			n = 153		
**Age, years, mean (±SD)**	70.5	(±12.7)	69.5	(±9.9)	70.8	(±13.6)	0.229	71.0	(±13.4)	0.236
**Gender, male, n (%)**	136	(60.7)	39	(67.2)	97	(58.4)	0.237	86	(56.2)	0.145
**Source of admission, n (%)**										
* Sugical*	170	(75.9)	48	(82.8)	122	(73.5)	0.156	113	(73.9)	0.175
* Medical + Others* * *^3^*	54	(24.1)	10	(17.2)	44	(26.5)		40	(26.1)	
**Comorbidities, n (%)**										
* Liver cirrhosis*	20	(8.9)	4	(6.9)	16	(9.6)	0.789	16	(10.5)	0.600
* Portal hypertension*	12	(5.4)	3	(5.2)	9	(5.4)	1.000	9	(5.9)	1.000
* Status post oesophageal bleeding*	9	(4.0)	2	(3.4)	7	(4.2)	1.000	7	(4.6)	1.000
* Hepatic encelopathy*	4	(1.8)	1	(1.7)	3	(1.8)	1.000	3	(2.0)	1.000
* Cardiac insufficiency NYHA IV*	42	(18.8)	9	(15.5)	33	(19.9)	0.464	31	(20.3)	0.432
* Chronic pulmonary disease*	55	(24.6)	10	(17.2)	45	(27.1)	0.133	42	(27.5)	0.124
* Chronic obstructive pulmonary disease (COPD)*	47	(21.0)	8	(13.8)	39	(23.5)	0.118	36	(23.5)	0.120
* Lung fibrosis*	3	(1.3)	0	(0.0)	3	(1.8)	0.570	3	(2.0)	0.563
* Terminal renal insufficiency*	24	(10.7)	6	(10.3)	18	(10.8)	0.916	18	(11.8)	0.772
* Steroid medication*	12	(5.4)	2	(3.4)	10	(6.0)	0.736	10	(6.5)	0.518
* Chemotherapy*	12	(5.4)	3	(5.2)	9	(5.4)	1.000	9	(5.9)	1.000
* Immunosuppression therapy*	7	(3.1)	3	(5.2)	4	(2.4)	0.379	4	(2.6)	0.396
* AIDS*	0	(0.0)	0	(0.0)	0	0	1.000	0	0	1.000
* Leukemia*	2	(0.9)	0	(0.0)	2	(1.2)	1.000	2	(1.3)	1.000
* Lymphoma*	5	(2.2)	0	(0.0)	5	(3.0)	0.331	5	(3.3)	0.326
* Metastasing cancer*	23	(10.3)	4	(6.9)	18	(10.8)	0.454	18	(11.8)	0.449
**Severity Scores, mean (± SD)**										
* SAPS II Admission*	61.6	±17.4	64.6	±18.0	60.5	±17.0	0.176	60.9	±17.0	0.232
* SOFA Admission*	9.5	±3.6	10.0	±3.3	9.4	±3.7	0.363	9.4	±3.7	0.395
* SOFA day before EOLD/death*	10.6	±3.7	11.1	±3.4	10.5	±3.7	0.312	10.4	±3.7	0.233
* SOFA 2 days before EOLD/death*	10.2	±3.4	10.7	±3.6	10.0	±3.5	0.218	10.1	±3.4	0.289
**ICU LOS, days, median (IQR)**	5.5	(2–16)	3	(1–8)	7	(3–22)	<0.001	7	(3–23)	<0.001
**Organ replacement, n (%)**										
* Ventilation*	201	(89.7)	51	(87.9)	151	(91.0)	0.250	137	(89.5)	0.165
			+5* ^4^	(8.6)						
* Tracheostomy*	50	(22.3)	7	(12.1)	43	(25.9)	0.029	40	(26.1)	0.028
* Dialysis*	143	(63.8)	37	(63.8)	106	(63.9)	0.993	96	(62.7)	0.888
* IABP*	49	(21.9)	20	(34.5)	28	(16.9)	0.005	23	(15.0)	0.002
* VAD*	22	(9.8)	7	(12.1)	15	(9.0)	0.504	15	(9.8)	0.631
* ECMO/ECLS*	14	(6.3)	1	(1.7)	13	(7.8)	0.122	13	(8.5)	0.119
* Vasopressors*	202	(90.2)	55	(94.8)	145	(87.3)	0.142	134	(87.6)	0.205
**Blood products, n, median (Min-Max)**	11.5	(0–225)	10	(0–125)	12	(0–225)	0.409	12	(0–225)	0.372
**Advance directive with living and therapeutic will, n, (%)**	21	(9.4)	6	(10.3)	15	(9.0)	0.768	14	(9.2)	0.791
**Advance directive with patient´s surrogate decision maker, n, (%)**	20	(8.9)	5	(8.6)	15	(9.0)	1.000	13	(8.5)	1.000
**Patients with an attorney during ICU stay,** **n, (%)**	90	(40.2)	16	(27.6)	74	(44.6)	0.023	69	(45.1)	0.021
**Documentation in PDMS special section** * **^5^,** **n, (%)**	67	(29.9)	2	(3.4)	65	(39.2)	<0.001	62	(40.5)	<0.001

EOLD = End-of-life-decision; DNR = Do-not-resuscitate order; WH/WDLS = Withhold/Withdraw-life-support order;

* ^1^ = between Patients with a DNR and those without an EOLD;

* ^2^ = between Patients with a WH/WDLS order and those without an EOLD; SD = Standarddeviation; LOS = length of stay; IQR = interquartile range; Min = Minimum; Max = Maximum;

* ^3^ = Including Dermatology and Emergency Room;

* ^4^ = patients ventilated in the process of cardiopulmonary resuscitation; IABP = intra-aortic balloon pump; VAD = ventricular assist device; ECMO/ECLS = extracorporal membrane oxygenation/life system;

* ^5^ = special section of patientś main chart in the Patient data management system (PDMS) for documentation of social history and family conferences.

Fifty-one (22.8%) of all admissions to the ICU were planned admissions, 126 (56.3%) were emergency admissions. Every patient with an EOLD had a DNR order. One hundred fifty-three (92.2%) of EOLD-patients also had a WH/WDLS order. The majority of EOLDs (73.4% for DNRs and 73.2% for WH/WDLS) was done during the normal working hours from 7 a.m. to 5 p.m., 9.0% of DNRs and 7.2% of WH/WDLS orders were done during the night from 10 p.m. to 7 a.m. For DNRs decision incidences did not differ on weekends with 8.4% DNRs [95% CI: 7.3–9.6%] versus 9.5% No DNRs [95% CI: 1.0–17.9%], and 16.6% [95% CI: 15.1–18.1%] versus 16.2% [95% CI: 9.1–23.3%] on weekdays (p = 0.717), respectively. Also for WH/WDLS orders differences in incidences were not significant to No EOLDs with 8.2% [95% CI: 6.3–10.1%] on weekends and 16.7% [95% CI: 14.5–18.9%] on weekdays (p = 0.535).

**Figure 2:**
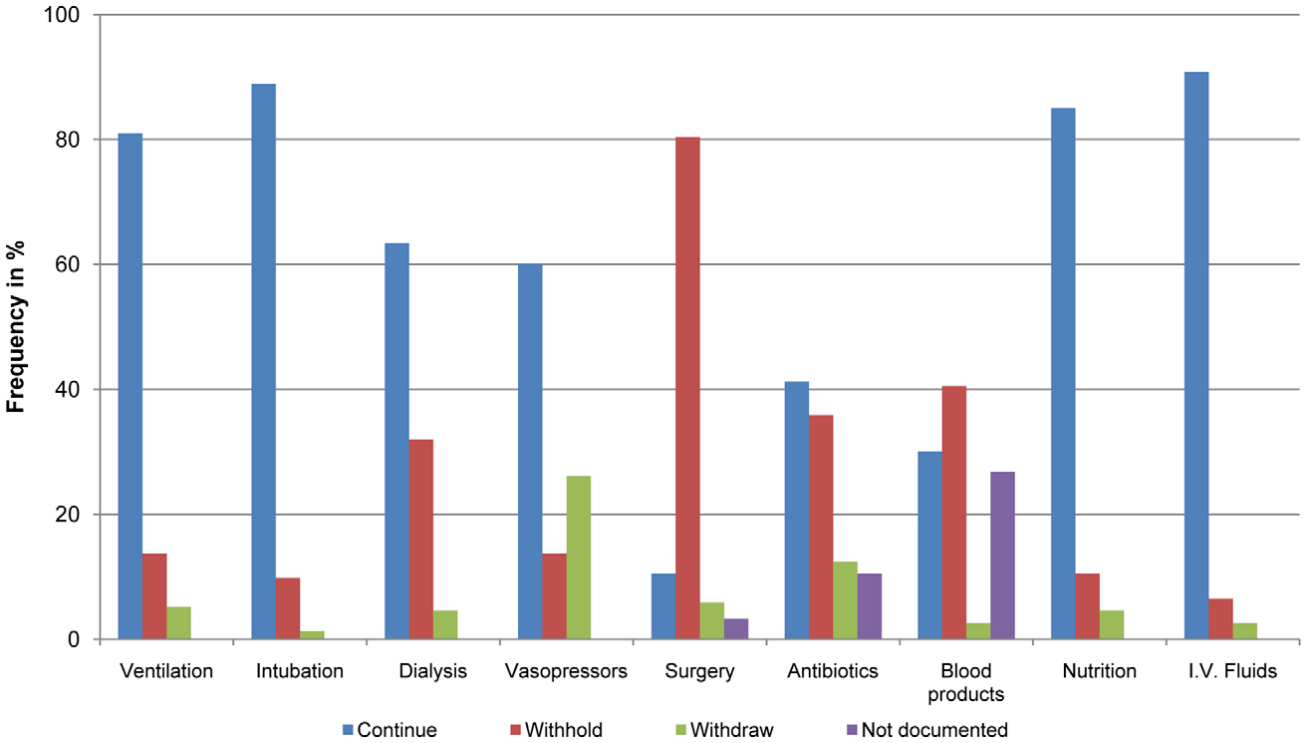
Practicing the end-of-life decision. Relative frequencies in percentage of continuing, withholding, or withdrawing of life sustaining treatment or the lack of documentation for the procedure regarding this entity in the end-of-life decision. Data are shown for the first withhold/withdraw life support order during the patientś stay on the intensive care unit.

For patients with a WH/WDLS order details of limiting life support are shown in Figure 2. Eight of 153 patients (5.2%) received an order to withdraw respiratory support mostly by decreasing the fraction of inspired oxygen to 21%. Just two patients (1.3%) were weaned to extubation. A maximum dosage for hemodynamic support with vasoactive drugs was defined in 75.2% of the cases.

One hundred thirty-one patients (91.2%) received the first WH/WDLS order the same time receiving the DNR order. In 22 patients (9.8%) there was a step by step approach from a DNR order to a WH/WDLS order. Frequencies for this approach did not change significantly from 13.8% (n = 87) to 12.7% (n = 79) of patients after September 1^st^ 2009 (p = 0.829). After a first WH/WDLS order 48 patients (31.4%) received additional orders for limitation of life support. Patient frequencies for this process stayed constant from 32.5% (n = 83) before to 30.0% (n = 70) after September 1^st^ 2009 (p = 0.737).

**Figure 3:**
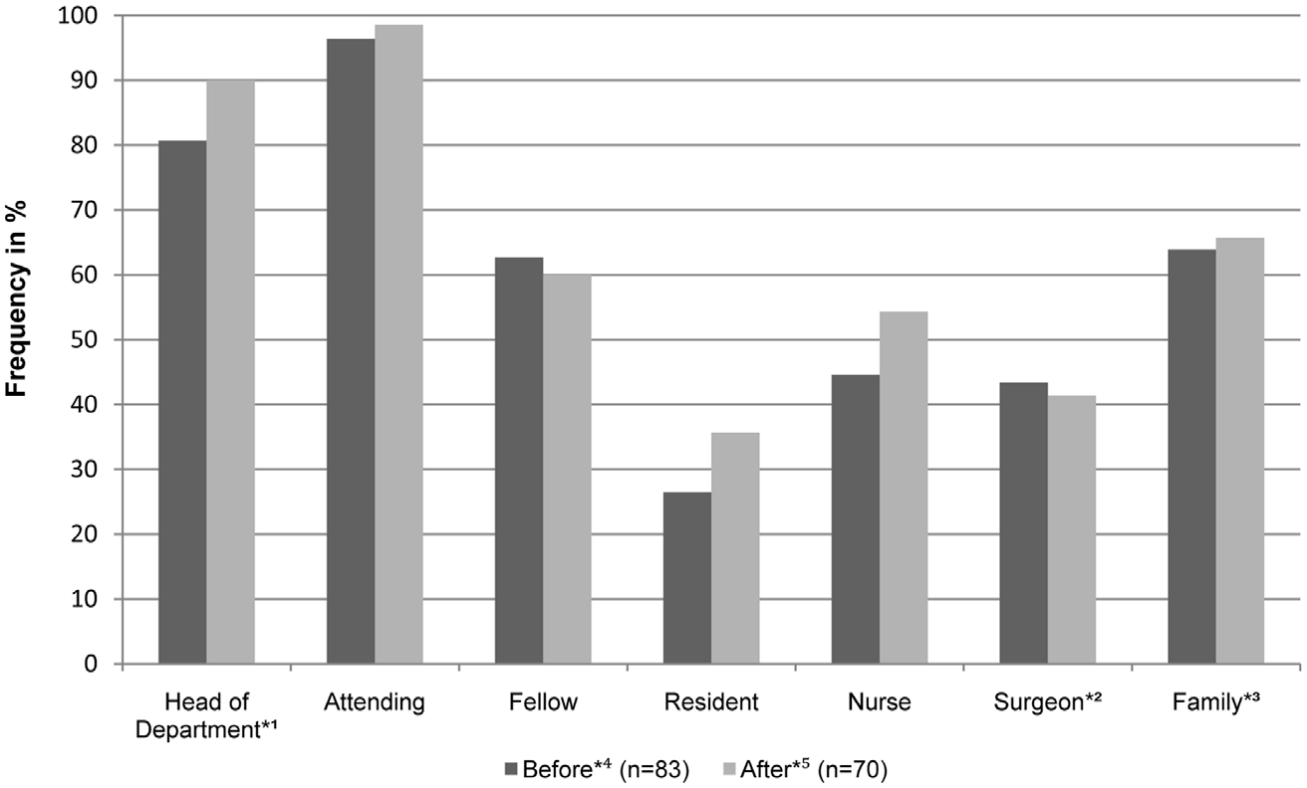
End-of-life decision makers for withhold and/or withdraw life support orders. Relative frequencies [%] of documented health care professionals and family members or patientś surrogate decision makers in the end-of-life decision process. *^1^ = the director of the Department of Anesthesiology and Intensive Care Medicine or her substitutes; *^2^ = the surgeon respective the physician primarily responsible for the underlying admission diagnosis; *^3^ = including the patient´s surrogate decision maker; Before*^4^ = period before 09/01/2009 with change of legislation; After*^5^ = period after 09/01/2009 with change of legislation.

Eight of 166 patients (4.8%) were informed about an EOLD and participated in the decision making process. Information of the patientś family or surrogate decision makers about an EOLD occurred in 147 (88.6%) of cases. In 102 (61,4%) of those cases the patientś family did actively participate in the decision making process. With regard to the new law no changes were noted in family information for DNRs [87.4% (n = 87) before vs. 89.9% (n = 79) after September 1^st^ 2009 (p = 0.611)] and for WH/WDLS orders [88.0% (n = 83) before vs. 91.4% (n = 70) after September 1^st^ 2009 (p = 0.484)]. The participation frequencies of the different decision makers for WH/WDLS orders with regard to September 1^st^ 2009 are shown in Figure 3.

About ten percent of ICU patients during the study period (10.3%) had some form of advance directive. Table 2 shows the differences in the incidences of advance directives and patientś attorneys between the periods before and after September 1^st^ 2009 for the different EOLD groups. Significant differences are noted for EOLD documentation (p<0.001) after September 1^st^ 2009.

Allowing for a so called wash-out period of six month after September 1^st^ 2009 for the policy to be implemented did not change our results in general. The only additional significant difference between the groups was a higher participation of residents in DNR- and in WH/WDLS-decisions after the new law (Tables S1 and S2).

## Discussion

Approximately three quarters of deaths on the ICU (74.1%) were preceded by an EOLD indicating that the process of dying was conscientiously taken into consideration and orchestrated by medical and nursing staff. Decisions on withholding or withdrawing therapeutic approaches were done patient-individually and irrespective of formal criterias.

EOLDs in the ICU follow a process of shared-decision making with participation of the different members of the medical team and the patients or their substitutes. This approach can be demonstrated for 24 hours every day of the week. An experienced ICU physician is involved in almost every EOLD and information and participation of the patient´s family occurs highfrequently.

During the observation period a law of advance directives was legislated in Germany for the first time considering a patient´s will that was written down in an advance directive as binding for physicians and the patientś surrogates. The process of differential decision making for DNRs and WH/WDLS orders was not affected by the new law. Also unaffected by the new legislation the prevalence of advance directives still resides around 10%. But the “advance-directives-law” has led to a significant improvement of documentation efforts of EOLDs on the ICU.

Once intubated we generally continue with ventilation and also with nutrition and fluid replacement. Entities that are deliberately withheld are those who cause additional harm to the organism like surgery. Although family members believe their relative is dying more comfortably while extubated it is known that there is an increased risk of patient´s distress during and after the process of discontinuing mechanical ventilation [20], [21]. As we and others found that death usually comes quickly after an EOLD we are obviously reluctant to wean patients from the ventilator most likely to reduce the risk of the patient experiencing any form of respiratory stress.

According to the international recommendations our end-of-life decision making process is conducted in a “shared” approach [9]. The attending as leader of the healthcare team is involved in almost every EOLD. Decisions are shared within the team of caregivers and the patient´s family participates in a very high proportion of EOLDs [1]–[4]. The incidences of decision maker participation in EOLDs look unaffected by the new legislation. Like the law-unaffected process of multistage decision making this indicates that we conduct EOLDs in a standardized process.

As in most other studies only a minority of patients could participate when an EOLD was instituted [7], [22]. Therefore the communication between clinicians and family is an integral tool to gain information about the patient´s most likely will [9], [23]. About 90% of families were informed of the EOLD respecting the fact that there are patients with no families nor anyone available to serve as a surrogate decision maker [24], [25]. But a family involvement is reported less frequently. This is in the range with data that exist for Central Europe [1], [3], [5]. One can question why obviously most families or patientś surrogates can be reached to be informed about the EOLD but not all of them are involved in the process of decision making. A possible explanation can be the fact that patients and therefore their surrogate decision makers can only refuse indicated treatment options but not request treatment that is not indicated.

In Germany as well as in other countries completion rates of advance directives are generally low [1], [4], [5], [14]–[16]. When German Federal Parliament passed the new law after a long and emotional debate we see no effects on the prevalence of advance directives in our study population. Advance directives have become known in public within recent years but people generally lack courage thinking about their own death [26]. They also might fear that due to rationing of resources in intensive care medicine patients with an advance directive are stigmatized and receive less aggressive care even when it would be appropriate to reverse critical illness. Interestingly the presence of an advance directive itself had no influence on a patient having an EOLD or not.

**Table 2:** Patientś advance directives and the new law.

	All			DNR			WH/WDLS		
	Before*^2^	After*^3^	p*^1^	Before*^2^	After*^3^	p*^4^	Before*^2^	After*^3^	p*^5^
	(n = 123)	(n = 101)		(n^ = ^87)	(n = 79)		(n^ = ^83)	(n = 70)	
**Advance directive with living and** **therapeutic will, n, (%)**	11 (8.9)	10 (9.9)	0.807	11 (12.6)	4 (5.1)	0.108	11 (13.3)	3 (4.3)	0.523
**Advance directive with patient´s** **surrogate decision maker, n, (%)**	10 (8.1)	10 (9.9)	0.644	9 (10.3)	6 (7.6)	0.537	9 (10.8)	4 (5.7)	0.393
**Patients with an attorney during ICU stay,** **n, (%)**	51 (41.5)	39 (38.6)	0.665	41 (47.1)	33 (41.8)	0.488	38 (45.8)	31 (44.3)	0.791
Documentation in PDMS special section*^6^, **n, (%)**	23 (18.7)	44 (43.6)	<0.001	23 (26.4)	42 (53.2)	<0.001	22 (26.5)	40 (57.1)	<0.001

p*^1^ = between Patients before and after 09/01/2009;

Before*^2^ = period before 09/01/2009 with change of legislation;

After*^3^ = period after 09/01/2009 with change of legislation;

p*^4^ = between Patients with a DNR before and after 09/01/2009;

p*^5^ = between Patients with a WH/WDLS order before and after 09/01/2009;

p*^6^ = special section of patientś main chart in the Patient data management system (PDMS) for documentation of social history and family conferences.

This is conflicting with recently published data showing that people with an advance directive were less likely to receive all possible care [27]. However in that study only 38.9% of the decedents died in hospital, 34.3% of them were nursing home residents and “most deaths were expected at about time they occurred” [27]. Obviously this cannot be generalized to ICU-patients with a high proportion of surgical cases like in our study. Moreover the mean age of the patients in our study is about 10 years lower. Focusing on studies that investigated advance care planning in conjunction with ICU-patients, there was no beneficial effect seen on patient care associated with advance directives [25], [28]–[30]. In another recently published survey about the role of advance directives in intensive care medicine in Austria Schaden, et al. questioned why “some colleagues still prefer to decide according to their own ethical concepts instead of honoring their patients wishes” [31]. But due to the missing difference between the EOLD and the No EOLD group in our study we can also speculate that the value of advance directives for this process is not that high as it is expected in public. In treating critically ill patients we are practicing medicine with a high burden of unpredictability, uncertainty and complex circumstances. Thus there are so many variables in critical care a patient really cannot oversee when he is planning his end-of-lifetime issues [32]. Therefore an EOLD that mostlikely reflects the patient´s will in those situations requires close communication and adequate documentation within the team of caregivers and the patient´s surrogate decision makers.

Adequate documentation and communication to family members of EOLDs is known to improve patientś and family outcomes, and increases transparency, reflection and teaching of intensive care therapeutic goals [33], [34]. However, it also requires increased time and health-care resources of the caregivers [35]. Our results indicate that documentation efforts and participation of residents in EOLDs have increased with the new law. While primarily aiming to strengthen the value of advance directives the new law has emphasized the importance of documenting discussions with family members to improve patient care. This finally culminated end of 2010 in defining “documentation of relatives meetings” as one of ten quality indicators in intensive care medicine in Germany [36].

### Conclusions

EOLD and end-of-life care has become an important part of intensive care medicine because many ICU-patients lack decision making capacity or an advance directive. Decision making regarding limitation of life prolonging therapy by DNR, withholding or withdrawing treatment options is performed by a team of experienced physicians shared in most cases with the patient´s surrogates and nurses. To avoid uncertainties between ICU staff and patients surrogates with regard to patientś supposed intentions close communication and standardized documentation is required to better understand the decision making process. Therefore, EOLD still remains a significant part of ICU profession and requires communicational skills to be trained. It cannot be regulated alone by legal restraints.

## Supporting Information

Table S1 **Patients’ advance directives and the new law (all with wash-out period).** Before*^1^ = period before 09/01/2009 with change of legislation; Wash-out*^2^ = period from 09/01/2009 with change of legislation until 03/01/2010; p*^3^ = between Patients before 09/01/2009 and wash-out period; After wash-out*^4^ = period from 03/01/2010 until 09/30/2012; p*^5^ = between Patients before 09/01/2009 and after the wash-out period; p*^6^ = between patients of the wash-out period and patients after the wash-out period; p*^7^ = special section of patientś main chart in the Patient data management system (PDMS) for documentation.
(DOC)

Table S2 **Patients’ advance directives and EOLDs with regard to the new law (DNR and WH/WDLS with wash-out period).** Before*^1^ = period before 09/01/2009 with change of legislation; Wash-out*^2^ = period from 09/01/2009 with change of legislation until 03/01/2010; p*^3^ = between Patients before 09/01/2009 and wash-out period; After wash-out*^4^ = period from 03/01/2010 until 09/30/2012; p*^5^ = between Patients before 09/01/2009 and after the wash-out period; p*^6^ = between patients of the wash-out period and patients after the wash-out period; p*^7^ = special section of patientś main chart in the Patient data management system (PDMS) for documentation.
(DOC)
