# Supplementary material for: Managing End-Of-Life Decision Making in Intensive Care Medicine – A Perspective from Charité Hospital, Germany
Source: PLoS One. 2012 Oct 1;7(10):e46446. doi: 10.1371/journal.pone.0046446 (PMC3462175; doi:10.1371/journal.pone.0046446)
Supplement: Table S1 — Patients’ advance directives and the new law (all with wash-out period). Before*1 = period before 09/01/2009 with change of legislation; Wash-out*2 = period from 09/01/2009 with change of legislation until 03/01/2010; p*3 = between Patients before 09/01/2009 and wash-out period; After wash-out*4 = period from 03/01/2010 until 09/30/2012; p*5 = between Patients before 09/01/2009 and after the wash-out period; p*6 = between patients of the wash-out period and patients after the wash-out period; p*7 = special section of patientś main chart in the Patient data management system (PDMS) for documentation. (DOC) [file pone.0046446.s001.doc]

**Table S1.** **Patients´ advance directives and the new law (all with wash-out period)**

|  | **All** | | | | | |
| --- | --- | --- | --- | --- | --- | --- |
|  | **Before*^1^** | **Wash out*^2^** | **p*^3^** | **After wash out*^4^** | **p*^5^** | **p*^6^** |
|  | **(n = 123)** | **(n = 57)** |  | **(n=44)** |  |  |
| **Advance directive with living and therapeutic will, n, (%)** | 11 (8.9) | 6 (10.5) | 0.786 | 4 (9.1) | 1.000 | 1.000 |
| **Advance directive with patient´s surrogate decision maker, n, (%)** | 10 (8.1) | 4 (7.0) | 1.000 | 6 (13.6) | 0.369 | 0.325 |
| **Patients with an attorney during ICU stay, n, (%)** | 51 (41.5) | 21 (36.8) | 0.625 | 18 (40.9) | 1.000 | 0.686 |
| **Documentation in PDMS special section*^7^ , n, (%)** | 23 (18.7) | 21 (36.8) | 0.010 | 23 (52.3) | <0.001 | 0.157 |
