# Supplementary material for: Managing End-Of-Life Decision Making in Intensive Care Medicine – A Perspective from Charité Hospital, Germany
Source: PLoS One. 2012 Oct 1;7(10):e46446. doi: 10.1371/journal.pone.0046446 (PMC3462175; doi:10.1371/journal.pone.0046446)
Supplement: Table S2 — Patients’ advance directives and EOLDs with regard to the new law (DNR and WH/WDLS with wash-out period). Before*1 = period before 09/01/2009 with change of legislation; Wash-out*2 = period from 09/01/2009 with change of legislation until 03/01/2010; p*3 = between Patients before 09/01/2009 and wash-out period; After wash-out*4 = period from 03/01/2010 until 09/30/2012; p*5 = between Patients before 09/01/2009 and after the wash-out period; p*6 = between patients of the wash-out period and patients after the wash-out period; p*7 = special section of patientś main chart in the Patient data management system (PDMS) for documentation. (DOC) [file pone.0046446.s002.doc]

**Table S2. Patients´ advance directives and EOLDs with regard to the new law** (DNR and WH/WDLS with wash-out period)

|  | **DNR** | | | | | | | **WH/WDLS** | | | | | |
| --- | --- | --- | --- | --- | --- | --- | --- | --- | --- | --- | --- | --- | --- |
|  | **Before*^1^** | **Wash out*^2^** | **p*^3^** | **After wash out*^4^** | **p*^5^** | **p*^6^** | **Before*^1^** | | **Wash out*^2^** | **p*^3^** | **After wash out*^4^** | **p*^5^** | **p*^6^** |
|  | **(n=87)** | **(n = 43)** |  | **(n = 36)** |  |  | **(n=83)** | | **(n = 38)** |  | **(n = 32)** |  |  |
| **Advance directive with living and therapeutic will, n, (%)** | 11 (12.6) | 3 (7.0) | 0.385 | 1 (2.8) | 0.109 | 0.621 | 11 (13.3) | | 3 (7.9) | 0.545 | 0 (0.0) | 0.033 | 0.245 |
| **Advance directive with patient´s surrogate decision maker, n, (%)** | 6 (6.9) | 1 (2.3) | 0.164 | 5 (13.9) | 0.756 | 0.087 | 9 (10.8) | | 1 (2.6) | 0.169 | 3 (9.4) | 1.000 | 0.325 |
| **Patients with an attorney during ICU stay, n, (%)** | 41 (47.1) | 17 (39.5) | 0.457 | 16 (44.4) | 0.844 | 0.819 | 38 (45.8) | | 17 (44.7) | 1.000 | 14 (43.8) | 1.000 | 1.000 |
| **Documentation in PDMS special section*^7^ , n, (%)** | 23 (26.4) | 19 (44.2) | 0.048 | 23 (63.9) | <0.001 | 0.113 | 22 (26.5) | | 19 (50.0) | 0.014 | 21 (65.6) | <0.001 | 0.230 |
| **Information/involvement, n, (%)** |  |  |  |  |  |  |  | |  |  |  |  |  |
| *Patient informed* | 6 (6.9) | 0 (0.0) | 0.177 | 2 (5.6) | 1.000 | 0.204 | 6 (7.2) | | 0 (0.0) | 0.175 | 2 (6.3) | 1.000 | 0.205 |
| *Patient involved* | 7 (8.1) | 0 (0.0) | 0.095 | 2 (5.6) | 0.726 | 0.204 | 7 (8.4) | | 0 (0.0) | 0.097 | 2 (6.3) | 0.734 | 0.205 |
| *Family informed* | 76 (87.4) | 40 (93.0) | 0.385 | 32 (88.9) | 1.000 | 0.458 | 73 (88.0) | | 36 (94.7) | 0.336 | 28 (87.5) | 1.000 | 0.402 |
| *Family involved* | 53 (60.9) | 27 (62.8) | 0.851 | 21 (58.3) | 0.841 | 0.646 | 52 (62.7) | | 24 (63.2) | 1.000 | 19 (59.4) | 0.831 | 0.808 |
| **Escalation of EOLDs, n, (%)** |  |  |  |  |  |  |  | |  |  |  |  |  |
| *Escalation of WH/WDLS* |  |  |  |  |  |  | 27 (32.5) | | 6 (15.8) | 0.078 | 15 (46.9) | 0.195 | 0.008 |
| *Escalation DNR -> WH/WDLS* | 12 (13.8) | 6 (14.0) | 1.000 | 4 (11.1) | 0.777 | 0.748 | 12 (14.5) | | 6 (15.8) | 1.000 | 4 (12.5) | 1.000 | 0.745 |
| **Decision makers, n, (%)** |  |  |  |  |  |  |  | |  |  |  |  |  |
| *Head of Department* | 68 (78.2) | 39 (90.7) | 0.091 | 30 (83.3) | 0.626 | 0.499 | 67 (80.7) | | 36 (94.7) | 0.055 | 27 (84.4) | 0.791 | 0.234 |
| *Attending* | 84 (96.6) | 42 (97.7) | 1.000 | 35 (97.2) | 1.000 | 1.000 | 80 (96.4) | | 38 (100.0) | 0.551 | 31 (96.9) | 1.000 | 0.457 |
| *Fellow* | 55 (63.2) | 24 (55.8) | 0.449 | 23 (63.9) | 1.000 | 0.371 | 52 (62.7) | | 21 (55.3) | 0.549 | 21 (65.6) | 0.831 | 0.465 |
| *Resident* | 22 (25.3) | 9 (20.9) | 0.665 | 17 (47.2) | 0.021 | 0.054 | 22 (26.5) | | 9 (23.7) | 0.825 | 16 (50.0) | 0.026 | 0.027 |
| *Nurse* | 37 (42.5) | 22 (51.2) | 0.454 | 18 (50.0) | 0.550 | 1.000 | 37 (44.6) | | 22 (57.9) | 0.240 | 16 (50.0) | 0.678 | 0.631 |
| *Surgeon* | 37 (42.5) | 16 (37.2) | 0.576 | 14 (38.9) | 0.841 | 1.000 | 36 (43.4) | | 15 (39.5) | 0.698 | 14 (43.8) | 1.000 | 0.809 |
| *Family* | 54 (62.1) | 27 (62.8) | 1.000 | 24 (66.7) | 0.685 | 0.491 | 53 (63.9) | | 24 (63.2) | 1.000 | 22 (68.8) | 0.668 | 0.801 |
